# Novel Surrogate Markers of CNS Inflammation in CSF in the Diagnosis of Autoimmune Encephalitis

**DOI:** 10.3389/fneur.2019.01390

**Published:** 2020-02-14

**Authors:** Jocelyn X. Jiang, Nicole Fewings, Suat Dervish, Alessandro F. Fois, Stephen R. Duma, Matthew Silsby, Sushil Bandodkar, Sudarshini Ramanathan, Andrew Bleasel, Bryne John, David A. Brown, Ming-Wei Lin

**Affiliations:** ^1^Department of Immunopathology, New South Wales Health Pathology-ICPMR, Westmead Hospital, Westmead, NSW, Australia; ^2^Department Clinical Immunology, Westmead Hospital, Westmead, NSW, Australia; ^3^Sydney Medical School, University of Sydney, Sydney, NSW, Australia; ^4^Centre for Immunology and Allergy Research, The Westmead Institute for Medical Research, Westmead, NSW, Australia; ^5^Westmead Research Hub, Westmead Institute for Medical Research, Westmead, NSW, Australia; ^6^Department of Neurology, Westmead Hospital, Westmead, NSW, Australia; ^7^The Children's Hospital at Westmead, Westmead, NSW, Australia; ^8^Neuroimmunology Group, Kids Neuroscience Centre, Children's Hospital at Westmead, Westmead, NSW, Australia; ^9^Department of Anaesthetics, Westmead Hospital, Westmead, NSW, Australia

**Keywords:** autoimmune encephalitis, cytokines, inflammation, surrogate markers, cerebrospinal fluid, CSF, diagnostic investigations

## Abstract

**Background:** Autoimmune encephalitis (AE) is an important cause of refractory epilepsy, rapidly progressive cognitive decline, and unexplained movement disorders in adults. Whilst there is identification of an increasing number of associated autoantibodies, patients remain with a high clinical probability of autoimmune encephalitis but no associated characterized autoantibody. These patients represent a diagnostic and treatment dilemma.

**Objective:** To evaluate routine and novel diagnostic tests of cerebrospinal fluid (CSF) in patients with a high probability of AE to attempt to identify better biomarkers of neuroinflammation.

**Methods:** Over 18 months (2016–2018), adult patients with a high clinical probability of AE were recruited for a pilot cross-sectional explorative study. We also included viral polymerase-chain-reaction (PCR) positive CSF samples and CSF from neurology patients with “non-inflammatory” (NI) diagnoses for comparison. CSF was examined with standard investigations for encephalitis and novel markers (CSF light chains, and cytokines).

**Results and Conclusions:** Thirty-two AE patients were recruited over 18 months. Twenty-one viral controls, 10 NI controls, and five other autoimmune neurological disease controls (AOND) were also included in the analysis. Our study found that conventional markers: presence of CSF pleocytosis, oligoclonal bands, anti-neuronal immunofluorescence, and magnetic resonance imaging (MRI) changes could be suggestive of AE, but these investigations were neither sensitive nor specific. Promising novel makers of autoimmune encephalitis were the CSF cytokines IL-21 and IP10 which may provide better delineation between viral infections and autoimmune encephalitis than conventional markers, potentially leading to more immediate diagnosis and management of these patients.

## Introduction

Autoimmune encephalitis (AE) is an important cause of unexplained movement disorders, rapidly progressive cognitive decline and refractory epilepsy (1, 2). Whilst a proportion of these patients have associated detectable anti-neuronal antibodies, there is currently no gold standard investigation to confirm the diagnosis (2).

A further clinical challenge are patients who clinically appear to have AE, but no associated autoantibody is identified (3, 4). Supportive findings indicating AE include the following conventional surrogate markers of neuroinflammation on CSF: oligoclonal bands, raised protein level, and pleocytosis (2, 5). Patients with antibody-negative or positive AE may not have elevations of these markers but still respond to a trial of immunomodulatory treatment (2, 6). Earlier initiation of treatment may result in better outcomes (3, 4). However, treatment of AE often involves significant immunosuppression (1, 6) and the decision to subject an individual to therapy is challenging if convincing objective evidence of an autoimmune etiology is lacking (1).

Novel surrogate markers including CSF light chains and CSF cytokines have been associated with CNS inflammation (7–13). Indirect immunofluorescence (IIF) on primate brain using CSF for anti-neuronal antibodies often reveals staining patterns not associated with a known antigen (non-specific IIF) and it is unclear whether these patterns are indicative of CNS autoimmunity.

A superior biomarker which more reliably differentiates CNS autoinflammation from other causes will assist clinicians commence treatment earlier. Our study examines a cohort of patients with high clinical suspicion of AE to identify biomarkers that might indicate this disease.

## Materials and Methods

### Recruitment of Patients

This is an exploratory study aiming to identify conventional or novel surrogate biomarkers of neurological inflammation associated with AE which provides class III evidence for the potential of the CSF cytokines IL21 and IP10 as biomarkers for AE. This study was approved by the Ethics Committee of Westmead Hospital (LNR/16/WMED/192) and written informed consent was obtained by all participants.

Patients at a single quaternary referral center in Western Sydney, Australia were prospectively recruited over 18 months between 2016 and 2018 (Figure e-1). Previously proposed diagnostic criteria for antibody negative AE (2) includes subacute onset of working memory deficits; altered memory status or psychiatric symptoms with new focal CNS findings; and seizures not explained by previous known seizure disorder paired with investigation findings of CSF pleocytosis (2); magnetic resonance imaging (MRI) features suggestive of encephalitis; and exclusion of alternative causes. Therefore, our inclusion criteria was based on the clinical grounds for suspicion of AE: refractory or multiregional seizures/epilepsy; rapidly progressive cognitive decline and unexplained movement disorders (1–6). Adult patients (16 years or older) with a high clinical suspicion of AE, as assessed by a neurologist, were enrolled in the study.

The decision for recruitment was based on clinical grounds, prior to lumbar puncture and before knowledge of subsequent results of investigations. If investigations results revealed an alternative diagnosis, patients were reclassified to the appropriate group i.e., infectious. Patients with an identified autoantibody associated with AE were classified as antibody positive (AbPAE) while those without antibodies were classified as antibody negative (AbNAE). To prevent information bias, negative results from CSF analysis did not exclude enrolled patients.

### Recruitment of Controls

Any enrolled patients diagnosed with a CNS viral infection through polymerase-chain-reaction (PCR) were included in the infectious control cohort. In addition, stored CSF samples classified as viral infection based on positive PCR results were included as viral controls. These samples were supplied as deidentified aliquots.

Non-inflammatory (NI) control CSF samples were obtained from patients undergoing large-volume lumbar puncture for “non-inflammatory” neurological disease (NIND) and from patients undergoing routine spinal anesthesia. Patients in the NIND group had a diagnoses of simple headache, idiopathic intracranial hypertension (IIH), or normal pressure hydrocephalus (NPH).

A disease control group consisting of patients with neuropsychiatric lupus, cerebral vasculitis, and multiple sclerosis were also included (OAND group).

### Sample Collection and Storage

CSF samples for AE and NI controls were collected in standard 10 mL CSF tubes. CSF for light chains and cytokine analysis were aliquoted from these samples and frozen at −80 degrees Celsius. Assays for CSF light chains and CSF cytokines were batched for analysis to minimize analytical variation.

### Assays

All investigations unless otherwise stated, were performed at ICPMR (NSW Health Pathology, Australia).

Patients underwent conventional investigations for AE including blood tests and lumbar puncture for collection of CSF (Figure e-1). Conventional serum and CSF studies were: isoelectric focussing for oligoclonal bands (Sebia Paris, France), indirect immunofluorescence (IIF) on primate brain (Inova San Diego, USA) and line blot (PCA-1, PCA-2, ANNA-1, ANNA-2, Ma-1, Ma2, Amphiphysin, CV2, CRMP5) for onconeural antibodies (Ravo Bettlach Switzerland) and a limbic encephalitis panel [NMDAR, LGI-1, CASPR2, GABA (B), AMPA1 and AMPA2] on HEK2 transfected cells (Euroimmun Lubeck, Germany), as well as voltage-gated potassium antibodies (VGKC) (performed by radioimmunoassay; Queensland Pathology, Royal Brisbane Hospital, Australia; kits from RSR Cardiff, United Kingdom). Confirmation of IgLON5 antibody was performed at Euroimmun, Lubeck Germany based on staining pattern on primate brain IIF in our laboratory.

The following tests were also performed on serum: anti-thyroid antibody (Siemens Munich, Germany) and thyroglobulin antibody (Siemens Munich, Germany); and CSF: microscopy and culture; protein (Siemens Vista Erlangen, Germany), anti-glutamic acid decarboxylase (GAD) antibodies (ELISA, SEALS Pathology, Prince of Wales Hospital, NSW Australia; RSR Cardiff, UK), and polymerase chain reaction (PCR) for viral infections: HSV (Artus Hamburg, Germany), VZV (in-house PCR assay), ENTV (in house PCR assay), and EBV by PCR (Ellitech Paris, France).

All conventional investigations were collected according to current practice and performed according to the usual procedures available at the receiving diagnostic laboratory.

CSF studies performed purely on a research basis were: CSF light chains (Freelite assay; Binding Site, Birmingham United Kingdom) and a broad panel of CSF cytokines (Milliplex; Merk Millipore Darmstadt Germany) using the magnetic multi bead array kits (MPHSCTMAG28SK17; MPHCYP3MAG63K01; MPHCYTOMAG60K03; MPHCYTP2MAG62K02. Cytokines tested were: IFN-γ, ITAC/CXCL11, IL-12p70, TNFα, CXCL9, IP-10/CXCL10 (Th1 cytokines); IL-13, IL4, IL5, TARC/CCL17, Eotaxin/CCL11 (Th2 cytokines); IL17a, IL-6, IL-8 (Th17 cytokines); and IL-1β, IL-21, IL-2, IL-23, IL-7, IL-10, BCA-1/CXCL13, GMCSF, GCSF (other cytokines). Kits were chosen based on maximum sensitivity for cytokine detection. Lower limits of detection of the cytokine assay are detailed in Table e-3. Samples for CSF light chains and cytokines were run as per assay kit instructions. CSF cytokines were run by two operators and in duplicate except when the sample amount was insufficient when it was run in singlicate (six AE patients).

Any additional clinically necessary investigations for diagnosis or management including MRI was performed. MRI reports included in our data as suggestive of neuroinflammation had features of hyperintensity, hippocampal swelling, or other signs of oedema. Reports including cortical dysplasia, mild involutional change or atrophy, and bleeding were not considered positive. MRI results were not available for NI and viral controls.

### Clinical Details

Clinical details for AE patients, NI, and OAND cohorts were collected by interviewing treating clinicians and verified through medical records. Clinical data for viral samples were not available.

### Statistical Analysis

Analysis of the surrogate markers examined in this study was performed using StataMP 13 and scatterplot figures of results were prepared using GraphPad Prism.

For continuous independent variables, univariate analysis using the Mann-Whitney *U*-test or Kruskal-Wallis test were performed to compare the various disease groups. Univariate logistic regression was performed for binomial and categorical variables. Heat map analysis of cytokines was performed using Morpheus (Broad Institute) to find cytokines of potential interest.

Significant findings were then combined in a multivariate logistic model to determine markers that were significantly and independently associated with disease group classification (AE vs. viral, NI, and OAND groups). These markers were then fitted to a predictive model and a receiver operating characteristic (ROC) curve created.

## Results

A total of 32 patients with a high clinical probability of AE were recruited. These were subdivided into nine AbPAE patients and 23 AbNAE patients. Ten NI controls, 24 viral controls, and five OAND were also included in the analysis (Figure e-1). Demographic details of recruited patients are described in Table 1. Demographic details of the viral controls were unavailable. Clinical and diagnostic details for the AbPAE and AbNAE groups are summarized in Table e-1. The antibodies detected in the AbPAE group were NMDA-R (3), GFAP (1), IgLon5 (1), LGI-1 (1), CASPR2 (1), Anti-ANNA1(Hu) (1), and Anti-GAD (1).

**Table 1:** Patient Demographic Details.

	**NI controls**	**OAND controls**	**AbPAE**		**AbNAE**		
				***P*** **(vs. NI)**		***P*** **(vs. NI)**	***P* (vs.** **antibody positive)**
Number of patients	10	4	9	n/a	23	n/a	n/a
Median age	54	46.5	37	0.98	44	0.4	0.2
Age range	17–81	19–60	15–58	n/a	18–73	n/a	n/a
Gender (M:F)	4:6	1:3	3:6	0.4	16:7	0.1	0.02

Most investigations were analyzed in over 90% of samples from the AE groups. Exceptions were CSF GAD (78%) and serum VGKC (59%).

Of the 10 NI controls, five were perioperative patients where only 1 mL of CSF was able to be collected. These samples were reserved for assessment of novel markers.

Two of the OAND controls had commenced immunosuppression at time of lumbar puncture: one cerebral lupus (methotrexate and mycophenolate) and one with cerebral vasculitis (pulsed methylprednisolone). However, both these patients required intensification of immunosuppression prior to remission.

Twenty-five PCR positive viral controls were included in this study. One was a recruited patient diagnosed with herpes simplex virus (HSV) positive on PCR. Twenty-four others were obtained from aliquoted stored samples and consisted of nine enterovirus (ENTV) positive samples, four HSV positive, three Epstein-Barr virus (EBV) positive, and eight varicella-zoster virus (VZV) positive samples. All ENTV samples were 500 μL in volume and were used for both CSF cytokine and CSF light chain analysis. There were only 200 μL of CSF for VZV, EBV, and HSV samples. Therefore, 5 HSV, 3 EBV, and 4 VZV samples were used for cytokine analysis and a further 4 VZV samples were used CSF light chain analysis. Thirteen viral samples also underwent IIF on primate brain.

### Conventional Markers

There was a trend for increased proportions of positive results in some conventional CSF markers of CNS inflammation in patients with AE and OAND compared to NI controls (Table 2) but this was not seen in all patients. Comparison of these markers with viral controls was not available.

**Table 2:** Conventional markers of CNS inflammation.

	**NI, *N* = 5**	**OAND, *N* = 5**	**AbAE, *N* =9**	**AbNAE, *N* = 23**		
	***n* (%)**	***n* (%)**	***n* (%)**	***p***	***n* (%)**	***P***
CSF mononuclear cells ≥ 5	0 (0%)	1 (20%)	2 (22%)	n/a	4 (17%)	n/a
CSF oligoclonal bands	0 (0%)	3 (60%)	6 (67%)	n/a	5 (22%)	n/a
CSF protein > 0.45 g/L	3 (60%)	2 (40%)	4 (44%)	0.5	9 (32%)	0.6
Neuronal IIF (any)	1 (20%)	2 (40%)	n/a	n/a	8 (35%)	0.4
MRI changes	n/a	4 (80%)	3 (33%)	n/a	5 (22%)	n/a

P-values are calculated as AbAE or AbNAE vs. all other control groups (NI, viral and OAND).

Two of 9 (22%) of AbPAE and 4/23 (17%) of AbNAE patients had evidence of CSF pleocytosis; >5 mononuclear cells. Six of 9 (67%) of the APAE and 4/23 (17%) of AbNAE high-risk patients had evidence of CSF oligoclonal bands. None of the NI group had CSF pleocytosis or oligoclonal bands and these markers were not able to be statistically analyzed.

Raised CSF protein (>0.45 g/L) was seen in 4/9 (44%) of the AbPAE and 9/23 (32%) of AbNAE groups but also in 3/5 (60%) of the NI group.

CSF neuronal IIF staining was observed in 8/23 (35%) of the AbNAE group but was also observed in 1/5 of the NI group, 1/13 (8%) of the viral controls with sufficient sample for testing (enterovirus only) and 2/5 (40%) of OAND controls. The viral sample with non-specific IIF staining was EBV positive on PCR however this was supplied as a deidentified aliquot and further clinical details could not be verified.

Three of 9 (33%) of the AbPAE group and 5/23 (22%) of the AbNAE group had non-specific changes on MRI indicative of neuroinflammation. No MRI results were available for viral or NI controls.

Therefore, whilst markers such as CSF oligoclonal bands, pleocytosis or presence of MRI changes may indicate an autoimmune process, these are not sensitive or specific (14, 15) enough for a reliable diagnosis.

### CSF Cytokines

**Figure 1:**
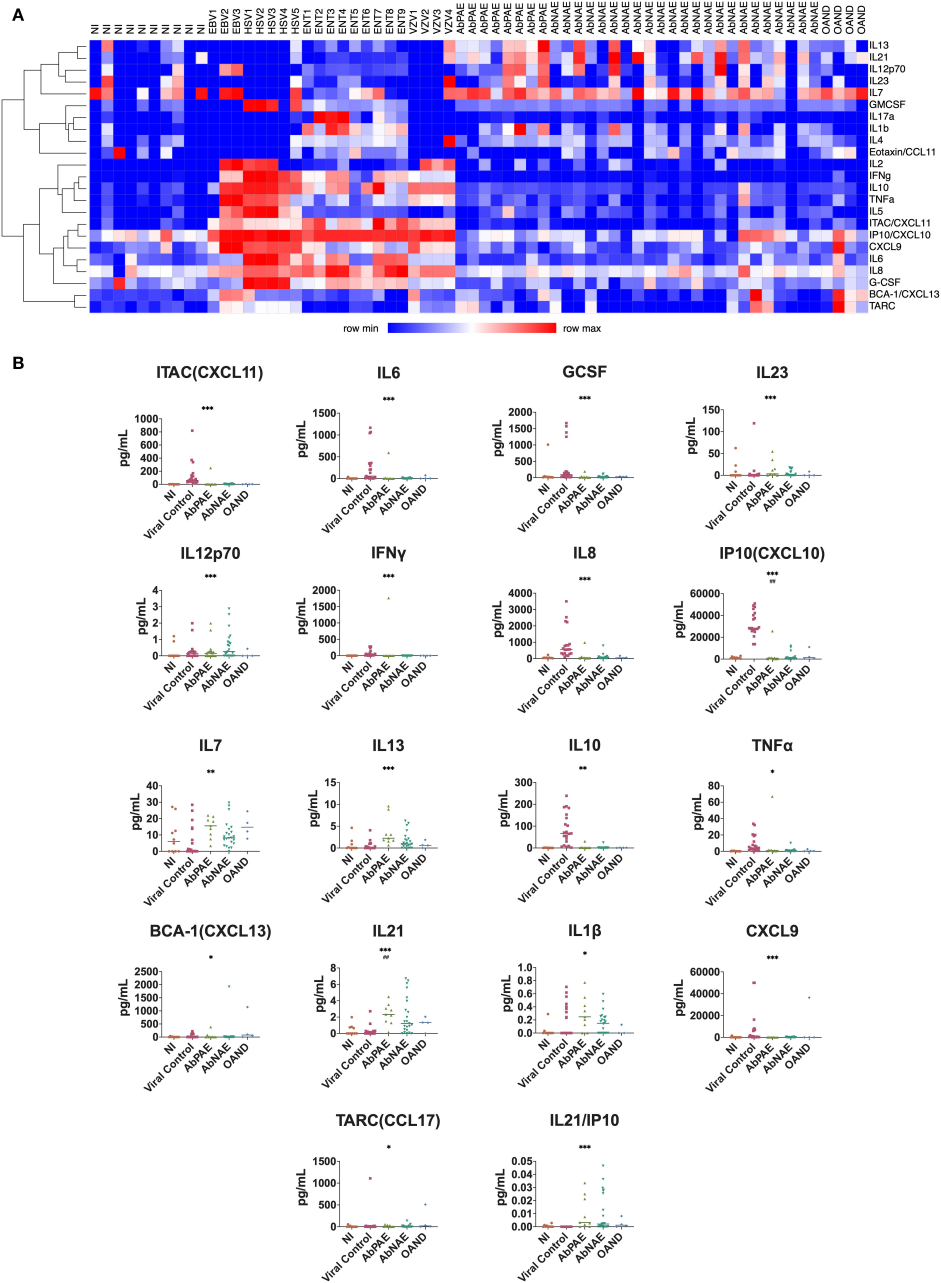
CSF cytokines. **(A)** Heat map cluster distinguishes viral from AbPAE and AbNAE encephalitis. Each column represents a participant. The X-axis identifies the cohort each participant belongs to, while the right-handed Y axis describes the corresponding cytokine. Increasing expression is depicted as increasing shades of red. Th1 and proinflammatory cytokines appear to be associated with viral infections, with the addition of IL7a, IL1b, and IL4 associated with enterovirus infections in this group. **(B)** Cytokines where a significant difference was found in univariate analysis of the autoimmune encephalitis group compared with a combined pool of NI, viral, and other autoimmune disease controls. Cytokines where the statistical significance was also seen in a univariate analysis are indicated with asterisks (* < 0.05, ** < 0.01, *** < 0.001, **** < 0.0001). Statistical significance seen in multivariate analysis are indicated with hatches (# < 0.05, *##* < 0.01, *###* < 0.001, *####* < 0.0001). Lines indicate medians. Table e-2 details the p-values of individual group comparisons. AbPAE, patients clinically high risk for autoimmune encephalitis who had identified associated antibodies; AbNAE, patients clinically high risk for autoimmune encephalitis without identified associated antibodies; NI, samples from patients either undergoing perioperative anesthesia or diagnosed with non-inflammatory neurological diseases; OAND, patients with other inflammatory neurological disease; EBV, Epstein Barr virus; VZV, varicella zoster virus; HSV, herpes simplex virus; ENT, enterovirus.

Heatmap cluster analysis revealed differential profiles of cytokine concentrations in patients with viral infections and NI controls compared to the combined AbPAE, AbNAE group, and OAND groups (Figure 1A).

There were no significant differences in cytokine levels between the AbPAE and AbNAE groups. Therefore, for statistical analysis, the AE patients were analyzed as one group when compared to NI and viral controls. The cytokines IL1b and IL12p70 were raised in the AE group when compared to the OAND group. Results of univariate analysis between individual groups are detailed in Table e-2.

Univariate analysis found that levels of IL21 (*p* = 0.0001), IL13 (*p* = 0.0002), IL12p70 (*p* = 0.0008) and IL7 (*p* = 0.009) were increased in the AE patients (Figure 1b) when compared to a combined cohort of normal, viral and OAND controls. As expected, Th1 related cytokines and other proinflammatory cytokines were elevated in viral controls.

**Figure 2:**
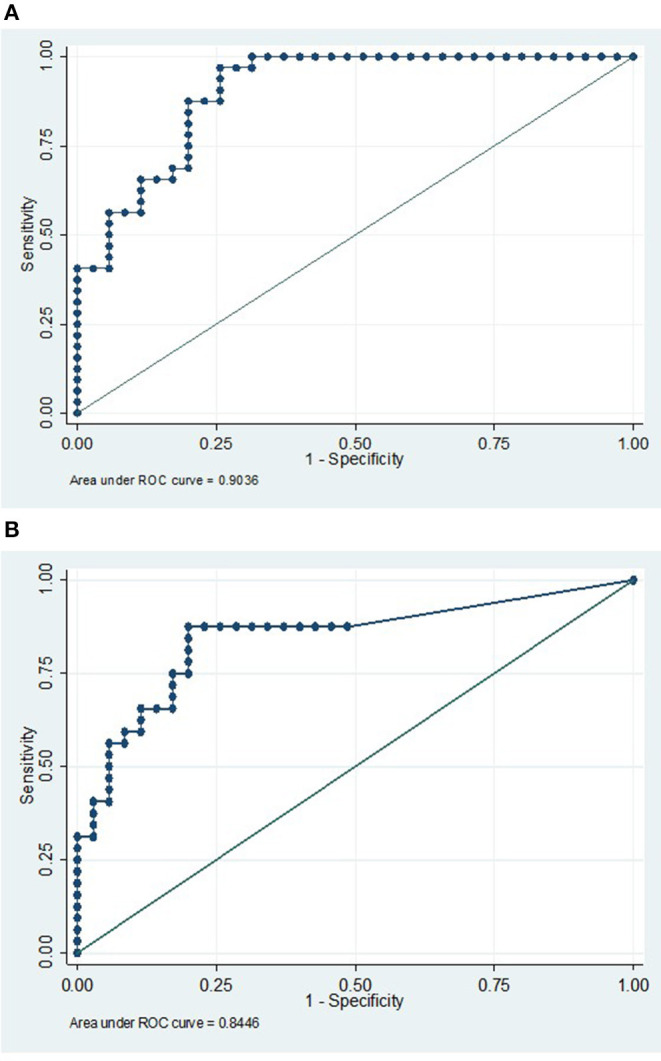
**(A)** IP10 and IL21 has a high sensitivity and specificity in discriminating AE from a combined control cohort. ROC curve analysis using a multivariate model with CXCL10/IP10 and IL21 in comparing a pooled AE cohort (compromising of both antibody positive and antibody negative groups) vs. a pooled viral control, NI cohorts, and OAND controls. AUC 0.90. **(B)** An IP10/IL21 ratio is a reasonably sensitive and specific single result that may differentiate the autoimmune encephalitis group from normal and viral controls. This ROC curve analysis uses a univariate logistic model with IL21/IP10 in comparing a pooled high risk for autoimmune encephalitis cohort (compromising of both antibody positive and antibody negative groups) vs. a pooled viral control and NI cohorts. AUC 0.84 AE, autoimmune encephalitis; NI, non-inflammatory controls; OAND, patients with other inflammatory neurological disease.

A multivariate logistic regression model was used to compare the combined cohort with a combined group of normal, viral, and OAND controls. Only IL-21 (*p* = 0.002) and CXCL10/IP-10 (*p* = 0.003) independently contributed to the model. A ROC curve constructed using this multivariate logistic regression model had an area under the curve (AUC) of 0.90 (Figure 2A). A ratio of IL21/IP10, in a univariate logistic regression model was also significant when compared a combined group of normal and viral controls (*p* = 0.01) with a ROC curve of 0.84 (Figure 1B).

### Other Novel CSF Markers

Only CSF lambda light chains were higher in both AE (*p* = 0.03) and viral control (*p* = 0.03) groups compared to NI controls. Kappa and lambda were both significant raised in OAND controls (*p* = 0.03 and 0.003, respectively). However, when the AE groups were compared to viral controls there was no statistical difference in CSF (kappa or lambda) light chains. Therefore, whilst CSF lambda may be a non-specific marker of neuroinflammation, it cannot be relied on to differentiate between AE from other differentials, such as viral infection.

## Discussion

The diagnosis of antibody-negative AE remains largely one of exclusion (2) and better biomarkers are required to assist with diagnosis to limit the potentially severe sequelae associated with treatment delays. Our study has demonstrated that selected CSF cytokines are promising biomarkers of AE, with or without characterized antibodies being present.

The most promising surrogate marker for AE is IL21 which was raised in both AbPAE and AbNAE groups. While the detected levels of IL-21 in the CSF were in the low range of the assay (0–6 pg/ml) they were significantly increased compared to viral and normal controls. Considering the short serum half-life of IL-21 (1–3 h) (16), this may represent sustained IL-21 production.

IL21 has many roles in B, CD8 T, and NK cell activation. In B cells, IL-21 acts as both an inhibitor and activator (17, 18). It stimulates apoptosis of B cells that become activated in the absence of T cell help (17, 18) but also stimulates B cell proliferation in the setting of T cell help. In combination with IL-4, IL21 has a significant role in switching B cells to IgG1 and IgG3 production (17–19). IL-21 also stimulates B lymphocyte-induced maturation protein transcription 6 (BLIMP6), which induces differentiation of B cells into long-lived plasma cells (17, 18). Therefore, IL-21, may contribute to autoantibody production in AE.

Antibodies associated with AE are continually being described. It is possible that patients diagnosed with antibody-negative AE may have an antibody that is yet to be discovered. Another consideration is that the finding of higher IL21 indicates a role in non-antibody mediated inflammation. IL21 down regulates FOXP3+ regulatory T cells leading to enhanced autoimmunity (17–19). In addition to being a T and NK cell activator, IL21 also critically regulates Th17 cell development, expansion, and function. With IL-7 or IL-15, IL-21 further enhances CD8+ T cell proliferation (17–19). It stimulates the proliferation of NK and NKT cells and enhances NK cytolytic function. These effects are demonstrated in anti-tumor models (17, 18) and may contribute to a predominantly cell-mediated autoinflammatory encephalitis.

The main mimic of AE is viral encephalitis which is an important consideration in the context of potential immunosuppression. The main cytokine indicative of viral infection in our study was IP10/CXCL10. IP10/CXCL10 is secreted in response to interferon gamma (20) which is produced as part of the Th1 response to viral infection. It is a chemoattractant for T cells, monocytes, natural killer (NK) cells and dendritic cells (20, 21). IP10/CXCL10 was raised in all viral infections included in our study. Other reports have associated IP10/CXCL10 with herpes and flaviviruses (22). However, this needs to be validated across a greater range of infections before it can be definitively used as a surrogate marker of infection.

Translating these findings into routine clinical practice, IL-21 and IP10 may contribute to the diagnostic armamentarium in the investigation of encephalitis, possibly helping to differentiate AE from conditions presenting in a similar fashion where immunosuppression may be harmful. A pragmatic way of comparing these values may be through an IL-21/IP10 ratio. In our cohort, this ratio had an excellent AUC on ROC curve analysis when AE was compared to NIND and viral controls, but this needs to be further validated.

Data available on CSF cytokines in this disease setting are limited and comparisons between studies are difficult because of heterogeneity in disease definitions and differences between cytokine detection platforms and their lower limit of detection, as well as kit manufacturers.

There is only one other study to our knowledge that examines CSF cytokine profiles in adults. This study examined CSF cytokines in 78 patients using a different platform manufacturer (Bio-Rad), including 20 with an autoimmune neurological disease (10). This study differed to ours in cohort with a significant proportion of patients with demyelinating disease (9/20) in autoimmune cohorts (excluded from our AE group) and patients with bacterial or tuberculosis CNS infections in their infectious cohort (9/38). They observed that MPO and IL8 was increased in cohorts with infectious and unknown etiology but did not find CXCL10/IP10 a significant marker of infection. They found IL-4, IL-10, IL-1R α, and IL1- β were higher in CSF of patients with immune-mediated disease (10). They did not examine IL21.

There is more literature available about CSF cytokines associated with AE in children, but it is unclear if these data are applicable to an adult population. One study (of children aged 28 days-14 years old) examined CSF cytokines (Bio-Rad kit) in viral encephalitis compared with NMDAR (four patients) encephalitis and found significant elevations in IL-6, IL7, and IL13 in the viral encephalitis group compared to the NMDAR encephalitis group (13), but did not examine IP10/CXCL10 or IL21. A second study examined the CSF of children (aged 2–14 years) with enterovirus encephalitis or NMDAR encephalitis and ADEM but found no significant differences in cytokine concentrations between these groups of patients. This study used cytokine kits from the same manufacturer as our study (Milliplex) but used kits under different catalog numbers to what we have used. Therefore, differences in findings for cytokine levels between this study and ours may reflect differences in children vs. adults or varying analytical sensitivities across different cytokine detection kits (11). A published review of CSF cytokines in children also found Th1 cytokines to be associated with viral encephalitis and CXCL13 and IL6 to be associated with NMDAR-antibody associated encephalitis and non-herpetic limbic encephalitis, respectively (12).

Our study did not find other novel potential novel markers useful in differentiating AE from NI or viral controls. In examining the literature, raised CSF free light chains (FLC) levels have been associated with neuroinflammation, however, the normal range is not well-established (7–9). Our results suggest it may be a better indication of general neuroinflammation rather than identifying a specific cause.

Currently utilized conventional markers are neither sensitive (1, 2, 23) nor specific (14, 15, 24, 25) enough for the diagnosis of AE (26). Whilst there were increased proportions of positive results in CSF pleocytosis and oligoclonal bands in our study in some AE patients, the presence of these markers have been described in infectious and/or neuroinflammatory states (14, 15, 24, 25). Similarly, the majority of cases of AE did not have detectable abnormalities on MRI (2). Elevated CSF protein was not a good indicator of neuroinflammation. There was a trend for an increased proportion of patients (35%) with any positive staining in IIF in the AbNAE group compared to one patient in the viral and NI groups, respectively. This may indicate that this finding may be useful but need to be examined further.

This study was limited by small patient numbers and limited CSF volumes, reflecting the rarity of this disease. There was difficulty in obtaining sufficient normal and viral control samples of CSF and there were pre-analytical collection issues in this study. The use of deidentified viral PCR positive CSF aliquots for controls meant clinical correlation was not possible. We did not check serum cytokines for patients in our cohort and comparison between CSF and serum cytokine levels in these patients should be a focus of further study.

Nevertheless, this is the first study, to our knowledge, to prospectively examine both conventional and novel markers of neuroinflammation in these groups of adult patients prior to immunosuppression. We have demonstrated the CSF cytokines CXCL10/IP-10 and IL-21 are potential differentiators of AE from viral encephalitis, particularly when there is no CNS specific autoantibody detected. These novel markers could have a future role to help expediate the decision to commence immunosuppression in this group of patients warranting their prospective validation in separate cohorts of AE patients.

## Data Availability Statement

The datasets generated for this study are available on request to the corresponding author.

## Ethics Statement

This study was approved by the Ethics Committee of Westmead Hospital (LNR/16/WMED/192) and written informed consent was obtained by all participants.

## Author Contributions

JJ: study design, patient recruitment, data extraction, cytokine assays, statistical analysis, drafting, and editing of manuscript. NF: cytokine assays, heatmap production, and editing of manuscript. SD: cytokine assays and editing of manuscript. AF, SRD, and MS: patient recruitment and editing of manuscript. SR and AB: initial study design and editing of manuscript. SB: assistance with assays and editing of manuscript. BJ: recruitment of controls and editing of manuscript. DB and M-WL: study design, patient recruitment, and editing of manuscript.

### Conflict of Interest

The authors declare that the research was conducted in the absence of any commercial or financial relationships that could be construed as a potential conflict of interest.

## Correction Note

A correction has been made to this article. Details can be found at: 10.3389/fneur.2026.1842731.
