# Correction: Novel surrogate markers of CNS inflammation in CSF in the diagnosis of autoimmune encephalitis

**DOI:** 10.3389/fneur.2026.1842731

**Published:** 2026-06-22

**Authors:** Jocelyn X. Jiang, Nicole Fewings, Suat Dervish, Alessandro F. Fois, Stephen R. Duma, Matthew Silsby, Sushil Bandodkar, Sudarshini Ramanathan, Andrew Bleasel, Bryne John, David A. Brown, Ming-Wei Lin

**Affiliations:** 1 Department of Immunopathology, New South Wales Health Pathology-ICPMR, Westmead Hospital, Westmead, NSW, Australia; 2 Department Clinical Immunology, Westmead Hospital, Westmead, NSW, Australia; 3 Sydney Medical School, University of Sydney, Sydney, NSW, Australia; 4 Centre for Immunology and Allergy Research, The Westmead Institute for Medical Research, Westmead, NSW, Australia; 5 Westmead Research Hub, Westmead Institute for Medical Research, Westmead, NSW, Australia; 6 Department of Neurology, Westmead Hospital, Westmead, NSW, Australia; 7 The Children's Hospital at Westmead, Westmead, NSW, Australia; 8 Neuroimmunology Group, Kids Neuroscience Centre, Children's Hospital at Westmead, Westmead, NSW, Australia; 9 Department of Anaesthetics, Westmead Hospital, Westmead, NSW, Australia

**Keywords:** autoimmune encephalitis, cytokines, inflammation, surrogate markers, cerebrospinal fluid, CSF, diagnostic investigations

In the published article, there was an error in Table 2 as published. “CSF monocytosis” in column should read “CSF mononuclear cells >5” The corrected Table 2 and its caption appear below.

**Table 2:** Conventional markers of CNS inflammation.

	NI, *N* = 5	OAND, *N* = 5	AbAE, *N* =9	AbNAE, *N* = 23		
	*n* (%)	*n* (%)	*n* (%)	*p*	*n* (%)	*P*
CSF mononuclear cells ≥ 5	0 (0%)	1 (20%)	2 (22%)	n/a	4 (17%)	n/a
CSF oligoclonal bands	0 (0%)	3 (60%)	6 (67%)	n/a	5 (22%)	n/a
CSF protein > 0.45 g/L	3 (60%)	2 (40%)	4 (44%)	0.5	9 (32%)	0.6
Neuronal IIF (any)	1 (20%)	2 (40%)	n/a	n/a	8 (35%)	0.4
MRI changes	n/a	4 (80%)	3 (33%)	n/a	5 (22%)	n/a

P-values are calculated as AbAE or AbNAE vs. all other control groups (NI, viral and OAND).

A correction has been made to **Supplementary Table e-2**.

**Table e-2:** Results of univariate analysis of cytokines between individual groups.

	AE vs. Viral		AE vs. NI		Viral vs. NI		AE vs. OAND		OAND vs. NI	
	Significant?	*P*-value	Significant?	*P*-value	Significant?	*P*-value	Significant?	*P*-value	Significant?	*P*-value
IL21	AE	0.0001	Y	0.0002	N	0.8	N	0.49	N	0.097
IL12p70	AE	0.009	Y	0.0164	N	0.2	AE	0.03	N	0.86
IL13	AE	0.0005	Y	0.0145	N	0.89	N	0.3	N	0.25
IL23	AE	0.0004	N	0.30	N	0.16	N	0.12	N	0.33
IL7	AE	0.001	N	0.2145	N	0.51	N	0.59	N	0.32
IP10	V	0.0001	N	0.2	Y	0.0001	N	0.37	N	0.9
IFN-γ	V	0.0001	N	0.36	Y	0.0001	N	0.7	N	0.86
IL10	V	0.0001	N	0.05	Y	0.0001	N	0.72	N	0.3
IL6	V	0.0001	N	0.7	Y	0.0001	N	0.66	N	0.33
IL8	V	0.0001	N	0.84	Y	0.0001	N	0.63	N	0.71
TNF-α	V	0.0001	Y	0.0093	Y	0.0001	N	0.96	N	0.093
BCA1/CXCL13	V	0.0007	N	0.3995	Y	0.0053	N	0.1	N	0.18
TARC/CXCL11	V	0.013	N	0.77	N	0.09	N	0.23	N	0.32
CXCL9	V	0.0001	N	0.86	Y	0.0001	N	0.8	N	0.54
IL5	N	0.12	Y	0.02	Y	0.006	N	0.26	N	0.86
IL17α	N	0.44	N	0.3	N	0.16	N	0.14	N	0.3
IL1β	N	0.5	Y	0.013	N	0.13	AE	0.038	N	1
GCSF	V	0.0001	Y	0.03	Y	0.042	N	0.7	N	0.27
IL4	N	0.9	Y	0.019	N	0.13	N	0.12	N	0.68
IL2	V	0.012	Y	0.019	Y	0.0012	N	0.09	Y	0.0001
ITAC	V	0.0001	N	0.49	Y	0.0001	N	0.49	N	0.21
Eotaxin	N	0.64	N	0.77	N	0.94	N	0.29	N	0.33
GMCSF	N	0.13	Y	0.018	Y	0.022	N	0.56	N	0.25

N, not significant; Y, significant; AE, significant and higher in the autoimmune encephalitis group; V, significant and higher in the VI group; AO, significant and higher in the OAND group. ^*^Levels of IL2 in OAND and Normal controls were both undetectable and therefore unable to be statistically compared.

Interpretation of *p*-values in the column under Viral vs. Normal/NI for TARC/CXCL11 and AE vs. Normal/NI for TNFα and IL2 were incorrect (*p*-values were correct but the interpretation was discordant).

The *p*-value in the column under Viral vs. Normal/NI for was incorrect due to a typo IL5 (the interpretation of the *p*-value was correct but was discordant).

The correct material statement appears below:

In the published article, there was an error where monocytosis is stated in the article it should read “pleocytosis” as this was not referring to an increased monocyte count but a mononuclear white cell count of >5.

A correction has been made to **Abstract**, *Results and conclusions*, Paragraph 1, sentence 2. This sentence previously stated:

“Our study found that conventional markers: presence of CSF monocytosis, oligoclonal bands, anti-neuronal immunofluorescence, and magnetic resonance imaging (MRI) changes could be suggestive of AE, but these investigations were neither sensitive nor specific.”

The corrected sentence appears below:

“Our study found that conventional markers: presence of CSF pleocytosis, oligoclonal bands, anti-neuronal immunofluorescence, and magnetic resonance imaging (MRI) changes could be suggestive of AE, but these investigations were neither sensitive nor specific.”

A correction has been made to **Introduction**, Paragraph 2, sentence 2. This sentence previously stated:

“Supportive findings indicating AE include the following conventional surrogate markers of neuroinflammation on CSF: oligoclonal bands, raised protein level, and monocytosis”

The corrected sentence appears below:

“Supportive findings indicating AE include the following conventional surrogate markers of neuroinflammation on CSF: oligoclonal bands, raised protein level, and pleocytosis”

A correction has been made to **Results**, *Conventional markers*, Paragraph 2, sentence 1. This sentence previously stated:

“Two of 9 (22%) of AbPAE and 4/23 (17%) of AbNAE patients had evidence of CSF monocytosis; >5 monocytes.”

The corrected sentence appears below:

“Two of 9 (22%) of AbPAE and 4/23 (17%) of AbNAE patients had evidence of CSF pleocytosis; >5 mononuclear cells.”

A correction has been made to **Results**, *Conventional Markers*, Paragraph 2, sentence 3. This sentence previously stated:

“None of the NI group had CSF monocytosis >5 or oligoclonal bands and these markers were not able to be statistically analyzed.”

The corrected sentence appears below:

“None of the NI group had CSF pleocytosis or oligoclonal bands and these markers were not able to be statistically analyzed.”

A correction has been made to **Results**, *Conventional markers*, Paragraph 6. This sentence previously stated:

“Therefore, whilst markers such as CSF oligoclonal bands, monocytosis or presence of MRI changes may indicate an autoimmune process, these are not sensitive or specific (14, 15) enough for a reliable diagnosis.”

The corrected sentence appears below:

“Therefore, whilst markers such as CSF oligoclonal bands, pleocytosis or presence of MRI changes may indicate an autoimmune process, these are not sensitive or specific (14, 15) enough for a reliable diagnosis.”

A correction has been made to **Discussion**, Paragraph 11, sentence 2. This sentence previously stated:

“Whilst there were increased proportions of positive results in CSF monocytosis and oligoclonal bands in our study in some AE patients, the presence of these markers have been described in infectious and/or neuroinflammatory states”

The corrected sentence appears below:

“Whilst there were increased proportions of positive results in CSF pleocytosis and oligoclonal bands in our study in some AE patients, the presence of these markers have been described in infectious and/or neuroinflammatory states”

The original article has been updated.

