# Supplementary material for: Novel Surrogate Markers of CNS Inflammation in CSF in the Diagnosis of Autoimmune Encephalitis
Source: Front Neurol. 2020 Feb 14;10:1390. doi: 10.3389/fneur.2019.01390 (PMC7034172; doi:10.3389/fneur.2019.01390)
Supplement: Supplementary file 1 [file Image_1.pdf]

Figure e-1

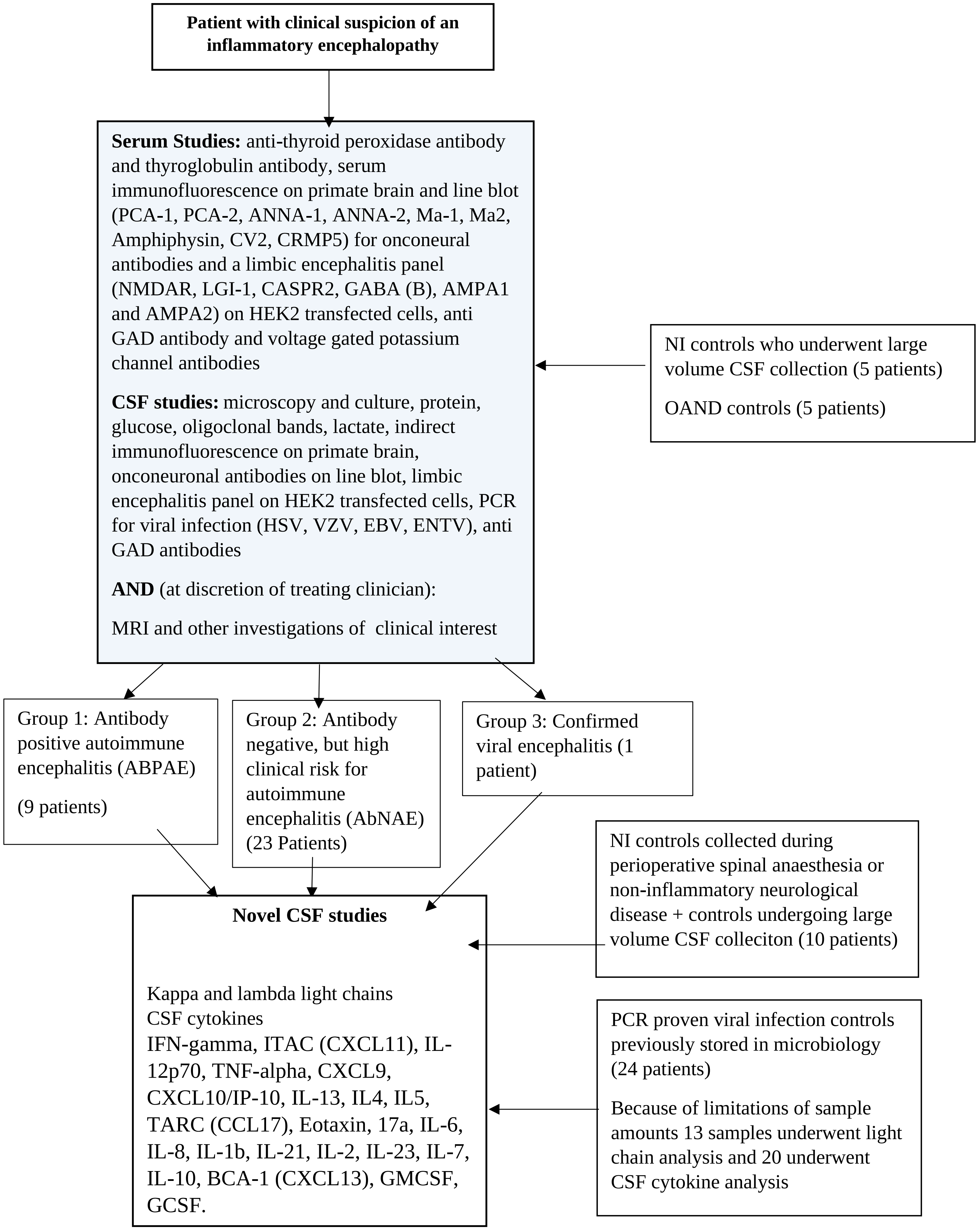
Patient with clinical suspicion of an inflammatory encephalopathy
Serum Studies: anti-thyroid peroxidase antibody and thyroglobulin antibody, serum immunofluorescence on primate brain and line blot (PCA-1, PCA-2, ANNA-1, ANNA-2, Ma-1, Ma2, Amphiphysin, CV2, CRMP5) for onconeural antibodies and a limbic encephalitis panel (NMDAR, LGI-1, CASPR2, GABA (B), AMPA1 and AMPA2) on HEK2 transfected cells, anti GAD antibody and voltage gated potassium channel antibodies
CSF studies: microscopy and culture, protein, glucose, oligoclonal bands, lactate, indirect immunofluorescence on primate brain, onconeuronal antibodies on line blot, limbic encephalitis panel on HEK2 transfected cells, PCR for viral infection (HSV, VZV, EBV, ENTV), anti GAD antibodies
AND (at discretion of treating clinician):
MRI and other investigations of clinical interest
NI controls who underwent large volume CSF collection (5 patients)
OAND controls (5 patients)
Group 1: Antibody positive autoimmune encephalitis (ABPAE)
(9 patients)
Group 2: Antibody negative, but high clinical risk for autoimmune encephalitis (AbNAE) (23 Patients)
Group 3: Confirmed viral encephalitis (1 patient)
Novel CSF studies
Kappa and lambda light chains
CSF cytokines
IFN-gamma, ITAC (CXCL11), IL-12p70, TNF-alpha, CXCL9, CXCL10/IP-10, IL-13, IL4, IL5, TARC (CCL17), Eotaxin, 17a, IL-6, IL-8, IL-1b, IL-21, IL-2, IL-23, IL-7, IL-10, BCA-1 (CXCL13), GMCSF, GCSF.
NI controls collected during perioperative spinal anaesthesia or non-inflammatory neurological disease + controls undergoing large volume CSF colleciton (10 patients)
PCR proven viral infection controls previously stored in microbiology (24 patients)
Because of limitations of sample amounts 13 samples underwent light chain analysis and 20 underwent CSF cytokine analysis

Supplement Figure 1: Recruitment criteria and of patients who entered the study, including patients and samples in the control groups, and all investigations conducted as part of the study.
