# Supplementary material for: Novel Surrogate Markers of CNS Inflammation in CSF in the Diagnosis of Autoimmune Encephalitis
Source: Front Neurol. 2020 Feb 14;10:1390. doi: 10.3389/fneur.2019.01390 (PMC7034172; doi:10.3389/fneur.2019.01390)
Supplement: Supplementary file 2 [file Table_1.docx]

**Table e-1:**

1. **Antibody-positive Patients: Clinical details and associated antibodies**

| Patient number | Clinical Presentation | Antibody associated |
| --- | --- | --- |
| **1** | 33F Refractory seizures requiring ICU admission | NMDA-R |
| **2** | 15F Severe headaches, atypical depression with psychosis, intermittent scotoma, cognitive decline | NMAD-R |
| **3** | 38F Transverse myelitis and encephalomeningial myelitis | GFAP |
| **4** | 18F Recurrent seizures, cognitive decline, optic neuritis | NMDA-R and Anti-MOG |
| **5** | 49M Insomnia, cold sensitivity, cerebellar ataxia | IgLON5 |
| **6** | 48F New temporal lobe seizures | LGI-1 |
| **7** | 55F New seizures, difficult to control | CASPR2 |
| **8** | 23M Difficult to control seizures, involuntary facial movements | Anti-ANNA 1 (Hu) |
| **9** | 58F Stiff Person’s syndrome | Anti-GAD |

1. **Clinical Details of AbNAE group**

| **Patient** | **Clinical presentation** |
| --- | --- |
| **1** | 69F, treatment resistant seizures |
| **2** | 18F recurrent seizures, facial twitching |
| **3** | 38M headache, recurrent/refractory seizures |
| **4** | 23M refractory seizures |
| **5** | 25M refractory seizures, psychosis, cognitive decline |
| **6** | 25M refractory seizures |
| **7** | 36F confusion, seizures, fatigue, short term memory loss |
| **8** | 22M Unusual behaviour, refractory seizures |
| **9** | 48M refractory seizures |
| **10** | 70M recurrent treatment resistant seizures, short term memory loss |
| **11** | 21F status epilepticus, acute memory decline, agitation, fevers, facial twitching, dysautonomia |
| **12** | 46F new onset treatment resistant seizures |
| **13** | 73M Cerebellar ataxia |
| **14** | 67M cerebellar ataxia, tremor, vitiligo |
| **15** | 32M rapidly progressive cerebellar syndrome |
| **16** | 66M rapid onset opsoclonus myoclonus; base of tongue tumour |
| **17** | 59M cerebellar ataxia |
| **18** | 26M movement disorder, facial dystonia |
| **19** | 70M cerebellar ataxia |
| **20** | 51M, rapid onset cognitive decline and movement disorder |
| **21** | 69M rapid cognitive decline, myoclonus |
| **22** | 69F rapid cognitive decline and psychosis |
| **23** | 38F agitation, rapid cognitive decline, lip smacking, unusual behaviour |
