# Supplementary material for: Novel Surrogate Markers of CNS Inflammation in CSF in the Diagnosis of Autoimmune Encephalitis
Source: Front Neurol. 2020 Feb 14;10:1390. doi: 10.3389/fneur.2019.01390 (PMC7034172; doi:10.3389/fneur.2019.01390)
Supplement: Supplementary file 3 [file Table_2.docx]

**Table e-2: Results of univariate analysis of cytokines between individual groups.**

|  | AE vs Viral | | AE vs NI | | Viral vs NI | | AE vs OAND | | OAND vs NI | |
| --- | --- | --- | --- | --- | --- | --- | --- | --- | --- | --- |
|  | Significant? | P-value | Significant? | P-value | Significant? | P-value | Significant? | P-value | Significant? | P-value |
| IL21 | AE | 0.0001 | Y | 0.0002 | N | 0.8 | N | 0.49 | N | 0.097 |
| IL12p70 | AE | 0.009 | Y | 0.0164 | N | 0.2 | AE | 0.03 | N | 0.86 |
| IL13 | AE | 0.0005 | Y | 0.0145 | N | 0.89 | N | 0.3 | N | 0.25 |
| IL23 | AE | 0.0004 | N | 0.30 | N | 0.16 | N | 0.12 | N | 0.33 |
| IL7 | AE | 0.001 | N | 0.2145 | N | 0.51 | N | 0.59 | N | 0.32 |
| IP10 | V | 0.0001 | N | 0.2 | Y | 0.0001 | N | 0.37 | N | 0.9 |
| IFN-γ | V | 0.0001 | N | 0.36 | Y | 0.0001 | N | 0.7 | N | 0.86 |
| IL10 | V | 0.0001 | N | 0.05 | Y | 0.0001 | N | 0.72 | N | 0.3 |
| IL6 | V | 0.0001 | N | 0.7 | Y | 0.0001 | N | 0.66 | N | 0.33 |
| IL8 | V | 0.0001 | N | 0.84 | Y | 0.0001 | N | 0.63 | N | 0.71 |
| TNF-α | V | 0.0001 | Y | 0.0093 | Y | 0.0001 | N | 0.96 | N | 0.093 |
| BCA1/CXCL13 | V | 0.0007 | N | 0.3995 | Y | 0.0053 | N | 0.1 | N | 0.18 |
| TARC/CXCL11 | V | 0.013 | N | 0.77 | N | 0.09 | N | 0.23 | N | 0.32 |
| CXCL9 | V | 0.0001 | N | 0.86 | Y | 0.0001 | N | 0.8 | N | 0.54 |
| IL5 | N | 0.12 | Y | 0.02 | Y | 0.006 | N | 0.26 | N | 0.86 |
| IL17α | N | 0.44 | N | 0.3 | N | 0.16 | N | 0.14 | N | 0.3 |
| IL1β | N | 0.5 | Y | 0.013 | N | 0.13 | AE | 0.038 | N | 1 |
| GCSF | V | 0.0001 | Y | 0.03 | Y | 0.042 | N | 0.7 | N | 0.27 |
| IL4 | N | 0.9 | Y | 0.019 | N | 0.13 | N | 0.12 | N | 0.68 |
| IL2 | V | 0.012 | Y | 0.019 | Y | 0.0012 | N | 0.09 | Y | 0.0001 |
| ITAC | V | 0.0001 | N | 0.49 | Y | 0.0001 | N | 0.49 | N | 0.21 |
| Eotaxin | N | 0.64 | N | 0.77 | N | 0.94 | N | 0.29 | N | 0.33 |
| GMCSF | N | 0.13 | Y | 0.018 | Y | 0.022 | N | 0.56 | N | 0.25 |

N, not significant; Y, significant; AE, significant and higher in the autoimmune encephalitis group; V, significant and higher in the VI group; AO, significant

and higher in the OAND group. *Levels of IL2 in OAND and Normal controls were both undetectable and therefore unable to be statistically compared.
