# Supplementary material for: Novel Surrogate Markers of CNS Inflammation in CSF in the Diagnosis of Autoimmune Encephalitis
Source: Front Neurol. 2020 Feb 14;10:1390. doi: 10.3389/fneur.2019.01390 (PMC7034172; doi:10.3389/fneur.2019.01390)
Supplement: Supplementary file 4 [file Table_3.docx]

Table e-3: Lower limits of detection for cytokines examined (as per kit protocols)

| Cytokine | Lowest limited of detection (pg/mL) |
| --- | --- |
| IL21 | 0.14 |
| IL12p70 | 0.15 |
| IL13 | 0.23 |
| IL23 | 3.25 |
| IL7 | 0.42 |
| IP10 | 8.6 |
| IFNg | 0.48 |
| IL10 | 0.56 |
| IL6 | 0.11 |
| IL8 | 0.13 |
| TNFα | 0.16 |
| BCA1/CXCL13 | 1.3 |
| TARC/CCL17 | 0.4 |
| CXCL9 | 19.2 |
| IL5 | 0.12 |
| IL17a | 0.33 |
| IL1β | 0.14 |
| GCSF | 1.8 |
| IL4 | 1.12 |
| IL2 | 0.19 |
| ITAC/CXCL11 | 1.25 |
| Eotaxin | 4.0 |
| GMCSF | 0.35 |
